# Regional mobility during the Covid-19 pandemic: Analysis of trends and repercussions using mobile phones data across the EU

**DOI:** 10.1016/j.cstp.2021.12.007

**Published:** 2022-03

**Authors:** Panayotis Christidis, Biagio Ciuffo, Michele Vespe

**Affiliations:** a European Commission, Joint Research Centre, Seville, Spain; b European Commission, Joint Research Centre, Ispra, Italy

**Keywords:** Mobility, Covid-19, Pandemic, Transport, European Union, Mobile phones, Regions, Telework, Non-pharmaceutical interventions

## Abstract

The analysis of mobile phones data at regional level in the EU reveals varying patterns in mobility trends during the Covid-19 pandemic. These depend on the temporal evolution of the pandemic in each EU Member State, the measures taken at local or national level to limit the growth of the pandemic, as well as the level of urbanization and type of economic activity in each region. During the first phase of the pandemic (March- April 2020) the decrease in mobility was in general uniform among regions in the same Member State, especially in Italy, Spain and France, where national level measures were adopted. A relaxation of the measures and a resulting rebound of mobility was evident during the summer period (July- August 2020). At the same time, a shift from urban to rural areas during the summer vacation period is evident, with especially touristic areas increasing the number of movements in the same Member State. The variance in mobility trends during the second wave of the pandemic (October- November 2020) was higher, a result of the predominantly local and regional level measures applied in each Member State. Those insights suggest a certain correlation between the level of mobility and the evolution of the pandemic at regional level. The association with high levels of Covid-19 prevalence is particularly strong in urban regions with high mobility levels.

## Highlights

• Analysis of mobile phones data across the EU.
• Varying patterns in mobility trends during the Covid-19 pandemic.
• Correlation between mobility and pandemic evolution at regional level.
• High Covid-19 prevalence is frequent in urban regions with high mobility levels.
• Limits on unsafe interactions more important than travel restrictions.

## 1 Introduction

The Covid-19 pandemic had a profound impact throughout the European Union (EU) and across the world. Apart from the obvious health dimension, the pandemic also led to a serious disruption of social and economic activities. Governments in many EU Member States and worldwide adopted measures aiming to decrease the risk of contagion. Such measures differed across countries and regions, as well as during the first and second wave of the pandemic and the period in between. While some Member States opted for a general limitation of activities, especially during the first wave, others opted for approaches based on awareness raising. Teleworking, where possible, was encouraged or even made obligatory. In many areas educational activities shifted to online modes during the first wave. Retail or entertainment activities in several cases had to adapt to timetable or capacity restrictions. Cross-national or –especially during the second wave- cross-regional or even intercity transport was limited by measures or lack of connections.

Either as a result of the measures applied or because of changing choices at individual level as regards social distancing, the evolution of the pandemic led to fluctuations in the levels of personal mobility. Most importantly though -since mobility is one of the many factors affecting the speed and spatial pattern of the evolution of the pandemic- the fluctuations in the levels of mobility can be a good predictor for the levels of contagion in the short term future (2–3 weeks). At regional level, the level of urbanization and the economic activity profile are factors that influence how mobility is affected by measures to limit the pandemic. As a result, there is a high variation as regards how each European region reacted in terms of changes in levels of mobility.

The case study presented here uses aggregate and anonymized movement data from a large number of Mobile Network Operators (MNOs) across EU Member States in order to monitor the evolution of mobility at regional level during 2020. The data were provided by European MNOs following a request by the European Commission for anonymized and aggregated mobile positioning data that would serve the following purposes in the fight against COVID-19 in order to (European Commission, 2020):

• “understand the spatial dynamics of the epidemics using historical matrices of mobility national and international flows;
• quantify the impact of physical distancing measures (travel limitations, non-essential activities closures, total lockdown etc.) on mobility, including the phasing out of such measures as relevant;
• feed epidemiological models, contributing to the evaluation of the effects of physical distancing measures on the reduction of the rate of virus spread in terms of reproduction number (expected number of secondary cases generated by one case);
• feed models to estimate the economic costs of the different interventions, as well as the impact of specific control measures on intra-EU cross border flows due to the epidemic.”

The contributions of MNOs in 23 European countries were collected on a daily basis and the resulting dataset was further processed through standardization and normalization techniques to ensure its compliance with the General Data Protection Regulation (GDPR) and to allow comparability across countries (Santamaria et al., 2020). The analysis presented here uses the dataset derived from the MNO data and explores the impact of Non-Pharmaceutical Interventions on mobility during the main phases of the pandemic with respect to the regional typology of European NUTS3 regions.

The main research question addressed is how the diversity of regional profiles within countries and across Europe influences the trends in mobility observed during the pandemic. The differences in the spatial and temporal patterns between urban and rural areas can reveal some underlying factors that influence the impact of policy measures and explain the role of regional mobility in the propagation of Covid-19. The novel aspect of this work is the combination of a dataset covering 23 countries for the whole of year 2020 with a geographic information analysis technique that allows a deeper insight into the factors that affect mobility.

## 2 Research context

The use of mobile phone data for the analysis of mobility is already a well-established approach (Bwambale et al., 2019). Mobile phone data offer suitable granularity for the monitoring of the pandemic since they allow a constant flow of information at high spatial resolution. Given the maturity of available methodologies, such data are often seen as a valuable input to researchers and policy makers (Kishore et al., 2020). Compared to other sources of data on mobility, mobile phones data provide timely information and allow a reliable identification of trip origins and destinations (Kang et al., 2020).

**Fig. 1:**
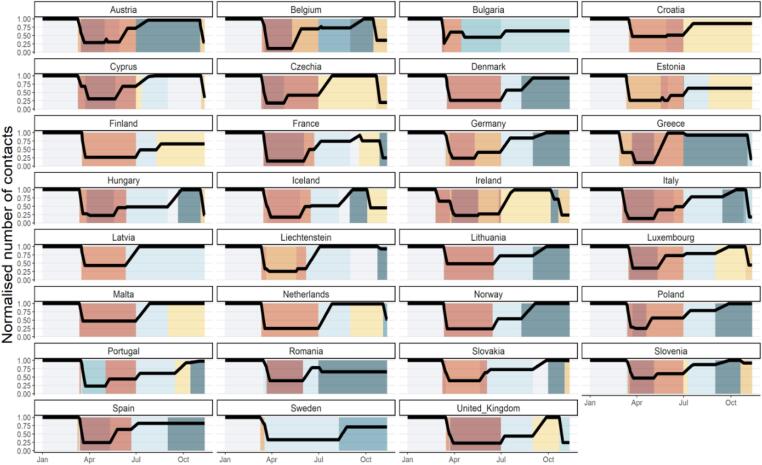
Effect of non-pharmaceutical interventions on the number of contacts in each Member State (source: ECDC (European Centre for Disease Prevention and Control, 2020), Fig. 7, p. 28).

Monitoring mobility at origin–destination level during the pandemic can be relevant for the design and evaluation of policy measures and especially non-pharmaceutical interventions. The type, duration and effectiveness of such measures differ significantly among countries and regions but also vary across time. A classification of the type of interventions applied in each EU Member State and their evaluation in terms of the impact on the number of contacts is available by ECDC (European Centre for Disease Prevention and Control, 2020) (Fig. 1). In the majority of cases, the impact on mobility appears to be in the same direction as the impact on the number of contacts. It is worth noting though that the level of the impact differs, with mobility indicators rebounding much slower than the number of contacts when measures are relaxed.

Work, education, retail and entertainment are the main drivers for mobility (Focas and Christidis, 2017), but differences in demographic and socio-economic characteristics affect the spatial and temporal patterns of demand at aggregate level (Fiorello et al., 2016). The evolution of mobility during the pandemic presented high variability among regions at European level, to a large extent due to the different repercussions that the epidemiological situation and the policy measures implemented on the social and economic structure of each region. Regions within countries responded heterogeneously to mobility reductions. During the first months of the pandemic, an association between the severity of the situation locally and the decrease in mobility was observed. Residents in areas with a high number of hospitalized Covid-19 patients or deaths related to the pandemic appear to have limited their exposure to risk. The high media visibility of the seriousness of the situation probably also contributed to the high levels of compliance with mobility restrictions (Pullano et al., 2020).

The average trip distance and overall transport demand fell significantly across Europe, although it is difficult to distinguish whether this is a consequence of policies or of voluntary behavioral changes (Christidis et al., 2021, Schlosser et al., 2020). Local and cultural factors have a strong role on how the population adheres to policy guidelines and is willing to adopt non-compulsory measures (Yabe et al., 2020). Policy interventions that led to a decrease in mobility have been associated with curbing the spread of Covid-19 (Gao et al., 2020), while rebounding mobility in many cases led to an increase (Chang et al., 2021). In this context, exploring the regional dimension of mobility during the pandemic can shed some light on the differences in the success of different types of interventions. Monitoring the evolution of mobility and Covid-19 prevalence at this level can also provide an input to the analysis of the factors that influence the spatial dimension of the pandemic.

## 3 Data and methods

The primary dataset used here is a combination of the data provided by the Mobile Network Operators (MNO) in 22 EU Member States plus Norway. No data were made available for Cyprus, the Netherlands, Luxembourg, Malta and Poland, The data definition and spatial resolution varies by operator, as does the time coverage of the data provided (Table 1).

**Table 1:** Data availability per EU Member State (plus Norway).

Member State	Number of NUTS3 regions	Earliest date available	Latest date available
Austria	36	31/01/2020	03/02/2021
Belgium	44	11/02/2020	03/02/2021
Bulgaria	29	01/02/2020	01/02/2021
Czechia	14	01/01/2020	03/02/2021
Denmark	11	02/02/2020	31/01/2021
Germany	401	01/01/2020	31/01/2021
Spain	59	01/02/2020	01/02/2021
Estonia	5	14/02/2020	31/01/2021
Finland	19	02/02/2020	31/01/2021
France	96	03/02/2020	01/08/2020
Greece	53	15/05/2020	04/02/2021
Croatia	21	01/02/2020	02/02/2021
Hungary	20	01/06/2019	03/02/2021
Ireland	8	01/02/2020	01/02/2021
Italy	116	01/01/2020	03/02/2021
Lithuania	10	01/02/2020	03/02/2021
Latvia	6	31/01/2020	02/02/2021
Norway	18	20/01/2020	31/01/2021
Portugal	25	01/02/2020	04/02/2021
Romania	42	15/04/2020	14/01/2021
Slovakia	8	03/02/2020	30/07/2020
Slovenia	12	01/02/2020	03/02/2021
Sweden	21	01/02/2020	03/02/2021

In order to convert the data into a common format that allows comparability, we performed a number of data processing actions:

• Aggregation of daily movements registered by each MNO at NUTS3 level: the granularity of the original data ranged from cell to municipality level
• Normalization of number of daily movements at NUTS3 level based on maximum total number of movements in each NUTS3 region: all data providers represent at least a 30% local market share and their data can be considered as representative of the population in the area they cover
• Aggregation of NUTS3 level movements based on origin and destination: internal in the same NUTS3 region, outwards to other NUTS3 region, inwards from other NUTS3 region
• Calculation of 3 mobility indicators (internal, outwards, inwards), indexed based on the maximum aggregate value per region and each of the 3 origin–destination combinations
• Calculation of 7-day moving averages to account for weekly variance
• Definition of urban classification using the GISCO NUTS 2016 definition (“GISCO, NUTS 2016 definition,” n.d.)
• For comparisons at aggregate level between the types of urbanization (urban, intermediate, rural), the indicators were weighted based on the 2019 population in each NUTS3 region (EUROSTAT, n.d.)

The original data provided by each MNO consisted of Origin-Destination matrices corresponding to the number of registered movements between origins i and destinations j during a specific time period. While the structure of the datasets produced by each provider was common, the spatial and temporal granularity varied considerably among operators, even within the same country. Differences include the spatial unit used, ranging from detailed cell tower level data to coarse administrative units, and the use of hourly or daily frequency for the registration of movements. The volume of the data submitted on a daily basis by each operator varied significantly as a result, ranging from less than 50 kilobytes to more than 30 Megabytes, resulting in a total data flow of about 200 Megabytes per day. Regardless of the specificities of each data source, each dataset maintains its internal consistency over time and space, a property that allows the calculation of relative changes in the indicator values. In order to make the indicators comparable across the EU, we aggregated the registered movements at NUTS3 level and calculated their daily totals.

The mathematical formulation of the three indicators follows a standardized structure for all spatial and temporal scales (Vespe et al., 2021):

(1) $$M_{Z}^{\mathrm{int}}\left(t\right)=\sum_{i\in Z}\sum_{j\in Z}{\mathrm{OD}}_{i,j}\left(t\right)$$

(2) $$M_{Z}^{\mathrm{inw}}\left(t\right)=\sum_{i\notin Z}\sum_{j\in Z}{\mathrm{OD}}_{i,j}\left(t\right)$$

(3) $$M_{Z}^{\mathrm{out}}\left(t\right)=\sum_{i\in Z}\sum_{j\notin Z}{\mathrm{OD}}_{i,j}\left(t\right)$$

where $M_{Z}^{\mathrm{int}}\left(t\right)$ , $M_{Z}^{\mathrm{inw}}\left(t\right)$ and $M_{Z}^{\mathrm{out}}\left(t\right)$ are the internal, inwards and outwards mobility indicators, respectively. Z is the geographic zone for which the calculation is made, which corresponds to the individual provinces (NUTS3 regions) covered in the analysis. ${\mathrm{OD}}_{i,j}$ is the number of registered movements with origin in a point i and destination in a point j, at time t. The aggregation of data at this level allowed a substantial compression of the resulting dataset, which for the analysis presented here was reduced to 21 Megabytes.

In order to explore possible correlations between mobility and the spread of the pandemic at regional level, we used data on reported cases from the Covid-19 European regional tracker (Naqvi, 2021a, Naqvi, 2021, Naqvi, 2021). The tracker compiles several epidemiological indicators at NUTS2 and NUTS3 level from a range of national and regional sources. We use the tracker’s data on the number of reported cases daily which –combined with the population data at regional level from EUROSTAT (EUROSTAT, n.d.)- allow the calculation of the 14-day notification rate of new Covid-19 cases for each region, one of the most frequently used indicators for monitoring the evolution of the pandemic.

## 4 Results

The detailed mobile phones data available allow the calculation of mobility indicators at regional level in a uniform format across the EU, even though the data structure and granularity may differ among operators. We used the dataset in two specific applications that are examples of how mobile phone data availability can serve in understanding the nexus between policy measures, mobility and the evolution of the pandemic:

From the mobility point of view, we explored how activity responded in each phase of the pandemic depending on the epidemiological situation and the policy measures in place;

From the epidemiological point of view, we investigated the potential correlation between mobility levels and the spread of Covid-19 at regional level.

### 4.1 Monitoring trends in mobility

**Fig. 2:**
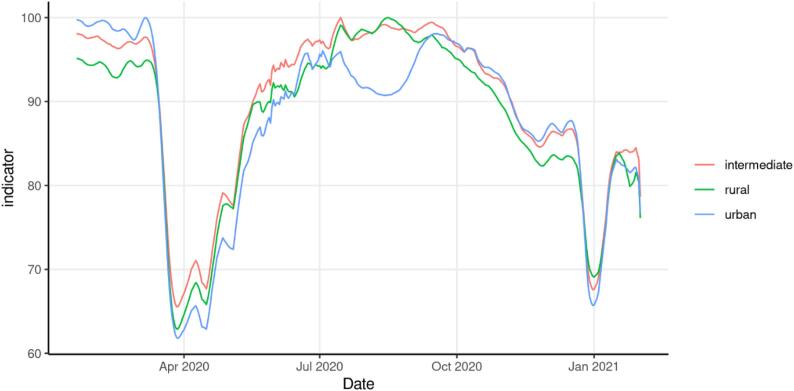
Evolution of average internal mobility by type of NUTS3 region, 7-day moving average, weighted by NUTS3 population (index: 100 = maximum mobility in NUTS3 region during the period).

Summarizing the results at the level of urban typology for the NUTS3 regions for which data are available reveals some visible patterns as regards the differences in mobility levels during the various phases of the Covid-19 pandemic (Fig. 2). The definition of NUTS3 regions varies considerably among EU Member States and amplifies the already important differences in terms of size and population, which leads to the well-known ‘boundary problem’ in spatial analysis. The use of indicators normalized based on the maximum observed value for each region neutralizes the potential distortions caused by the spatial definition (which would cause, for example, smaller regions to appear as having a larger share of inter-regional trips). At the same time, comparing the indicators in terms of regional typology allows an aggregation based on a combination of population density and economic activity, which can be useful for the interpretation of changes in mobility.

A pronounced reduction of individual mobility can be perceived starting in the second half of March 2020 and reaching the minimum levels of mobility on average about mid-April. While initially the decrease in mobility was practically the same for all three types of urbanization level, at the minimum -and for the entire period afterwards- the reduction in mobility was stronger in NUTS3 regions classified as predominantly urban. This can be probably explained by the difference in the type of economic activities in the three types of urbanization. A larger number of jobs in urban areas would be suitable for a shift to teleworking rather than in less urbanized zones (Soler et al., 2021). Retail and entertainment activities -which generate more transport activity in urban regions compared to non-urban areas- were also limited during the first phase in most Member States. In addition, risk aversion towards the use of public transport may have led to higher trip avoidance rates in urban areas.

With the intensity of the pandemic receding after May, accompanied by the relaxing of measures and citizen behavior, the mobility indicators recovered to comparable levels –about 90%- to those before the pandemic for all types of regions. Nevertheless, a reversal of the recovery trend can be observed in urban areas during the summer period. Holidays in educational activities and summer vacation for a large share of the labor force led to a noteworthy reduction of mobility indicators. In contrast, rural and intermediate regions continued their trend of recovery and actually reached –on average- the maximum level of the mobility indicator for year 2020. This indicates the considerable flow of residents in the major urban centers towards the main tourism and holidays destinations within each country.

After September 2020, the emergence of a second wave of the pandemic and the subsequent implementation of new policies can be reflected in a new shift of mobility indicators downwards. Measures taken during this period tended to be of a local or regional level, as opposed to country-wide ones, as was the norm during the first wave.

The trend was interrupted in the end of December/ beginning of January, with a marked decrease of the mobility indicator in all three groups. This was the result of the combination of the holiday period on one hand (which decreased the overall demand for mobility) and the emergency measures implemented in several Member States, on the other. The latter aimed at limiting the number of social interactions during the period in order to avoid spreading the disease even further (apparently unsuccessful, as in most Member States the holiday period probably triggered a new wave). The relative impact for each regional profile differs again, with urban areas registering a stronger decrease, an observation that possibly indicates a certain movement to rural areas during the holiday period. The downward trend of the mobility indicator continued in January, without however reaching the minimums of March 2020, even though the epidemiological situation was comparably worse in many EU Member States.

**Fig. 3:**
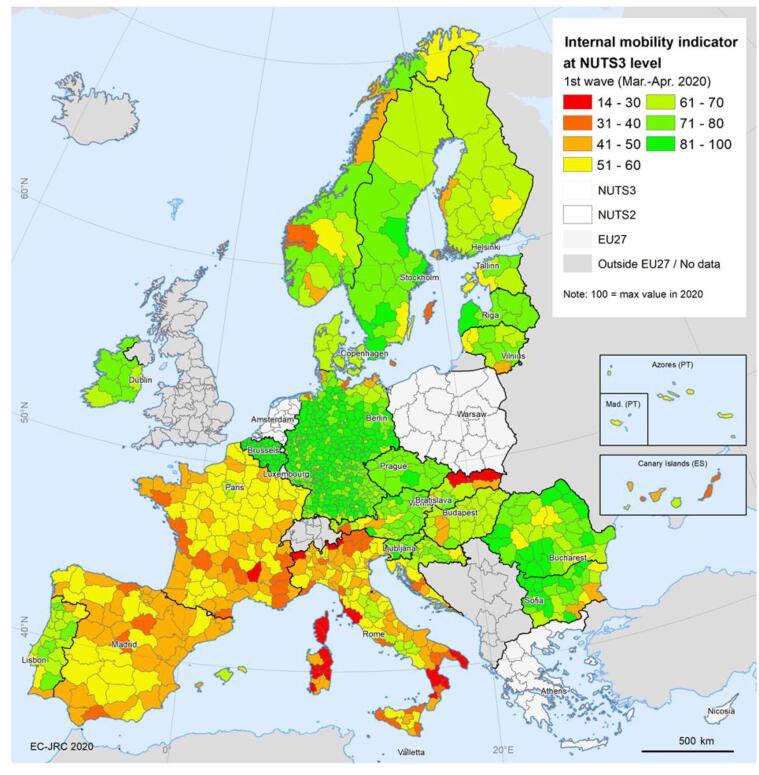
Average internal mobility by NUTS3 region, 1st wave of pandemic (March – April 2020); index: 100 = maximum mobility in NUTS3 region during year 2020.

The countries in the EU that were hit the hardest during the first wave of the pandemic were Italy, Spain and France. They were also the first Member States that introduced strict measures that limit mobility, such as stay-at-home enforcement and closure of non-essential activities. The combination of those measures with the generalized perception of high risk of contagion led to a steep decrease of mobility across all regions in these countries (Fig. 3). It is interesting to note that there was high regional variation within each of those countries as regards the level of decrease. For Italy and Spain, the major urban areas registered stronger decreases than the rest, but Paris showed a much more limited decrease than the rest of France. In contrast, for most other countries for which data are available the decrease in mobility was more limited (on average 20% lower than normal levels) and relatively uniform across all regions.

**Fig. 4:**
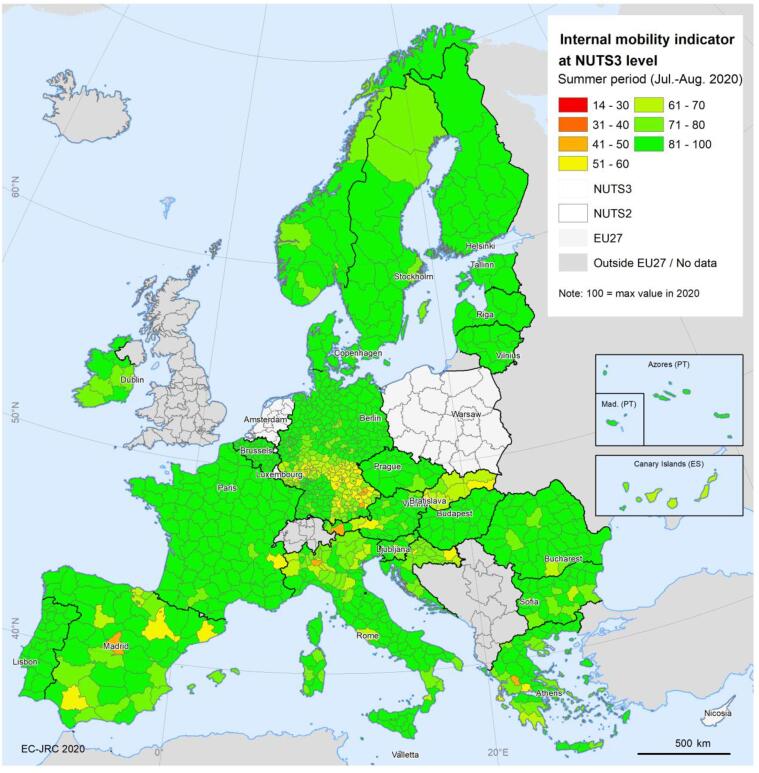
Average internal mobility by NUTS3 region, Summer period (July – August 2020); index: 100 = maximum mobility in NUTS3 region during year 2020.

During the summer period (July- August 2020) the level of mobility recovered in all Member States and Norway (Fig. 4). There was, however, a mismatch between the trends in urban areas as opposed to non-urban areas. The holiday period resulted in a large number of citizens leaving urban centers towards holiday destinations, to a large extent within the same Member State (given the overall uncertainty and the limited air travel options). This tendency was particularly visible in France, Spain and Italy, where the high population urban areas showed a marked decrease in mobility indicators, while traditional holiday destinations and non-urban areas in general maintained high levels (increased by the inflow of visitors from the same Member State). Most Member States had a mixed pattern of changes in mobility indicators at regional level.

**Fig. 5:**
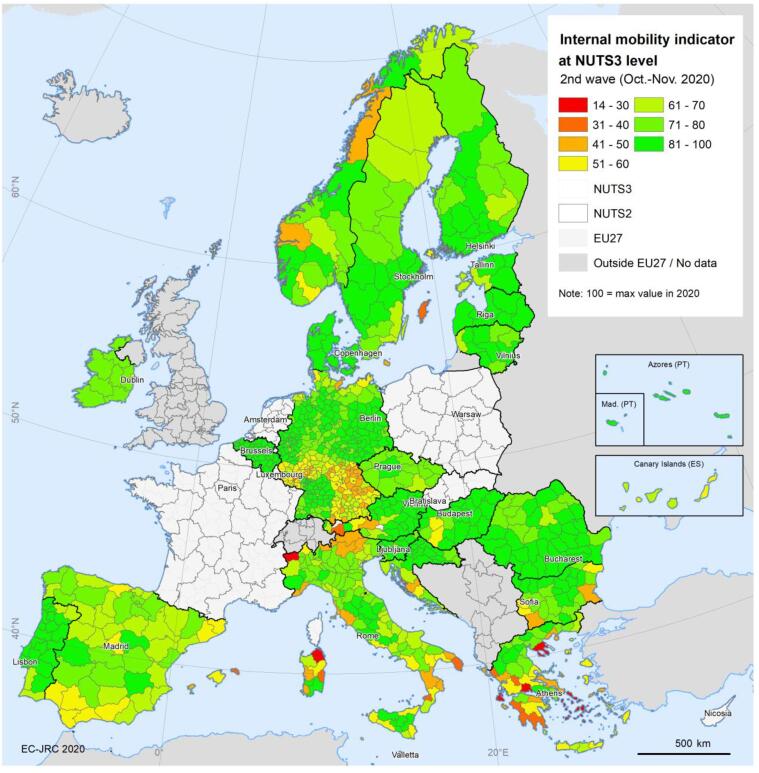
Average internal mobility by NUTS3 region, 2nd wave of pandemic (October – November 2020); index: 100 = maximum mobility in NUTS3 region during year 2020.

A third phase of the pandemic in terms of regional mobility is visible during October and November 2020, when a second wave of the pandemic emerged. In terms of measures adopted, the main difference with the first wave was that restrictions in most Member States were applied at local and regional level (as opposed to country-level). As a result, a high variability at both Member State and regional level can be observed (Fig. 5).

**Fig. 6:**
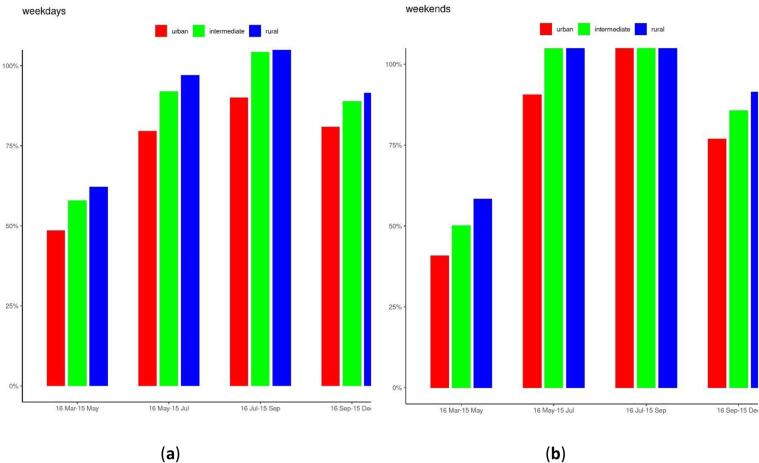
Evolution of mean mobility level by period (weighted by regional population; as % of mean mobility indicator in period 1st of January to 15th of March 2020): (**a**) weekdays (Monday to Friday); (**b**) weekends (Saturday and Sunday).

Another diverging pattern among regional mobility profiles can be observed in the variation across working and non-working days. Fig. 6 summarizes the evolution of the mobility in each of the three regional profiles, on weekdays and weekends across four characteristics periods in 2020. Comparing to a reference period before the pandemic (1 January to 15 March 2020), the relative ranking of the strength of the impact on mobility among regional profiles is maintained, with urban areas being in all cases the most affected, both on weekdays and on weekends. Mobility levels on weekends were lower than the ones on weekdays during the first wave of the pandemic (here labeled as 16 March- 15 May 2020) for all three profiles, but are higher in the following two period (from 16 May to 15 September). In fact, in non-urban areas weekend mobility was higher than during the reference period, probably due to the overall relaxation of measures, the better weather conditions and – especially during the summer period- increased mobility during the holiday period. After the 15th of September the relative difference from their reference levels was comparable for weekends and weekdays in all three regional profiles.

The outwards and inwards mobility indicators tend to be symmetrical on a weekly basis for most part of the year. The number of movements leaving a NUTS3 region during a weekday is normally compensated by the number of movements entering the same region. This effect can be attributed to the large share of commuting trips between neighbouring regions, for which a return trip usually takes place within the same day. Return trips during the weekends may show a longer lag, since they may involve an overnight (or longer) stay at the destination. The influence of this duration is already reflected in the difference in the mobility patterns between weekdays and weekdays (Fig. 6), with mobility levels in non-urban destinations being systematically higher during weekends than weekdays. The weekend effect is balanced out when mobility indicators are measured on a weekly basis by applying a 7-day moving average during most of the year. The longer stays in holiday destinations during the summer do –however- produce a temporary lag between the inwards and outwards indicators. Urban regions present a higher outwards mobility level in the beginning of the summer period which is compensated by a higher inwards mobility level at the end. For rural areas, the trend is opposite, since they tend to be the main holiday destinations. In either case though, the two indicators returned to similar levels in September.

**Fig. 7:**
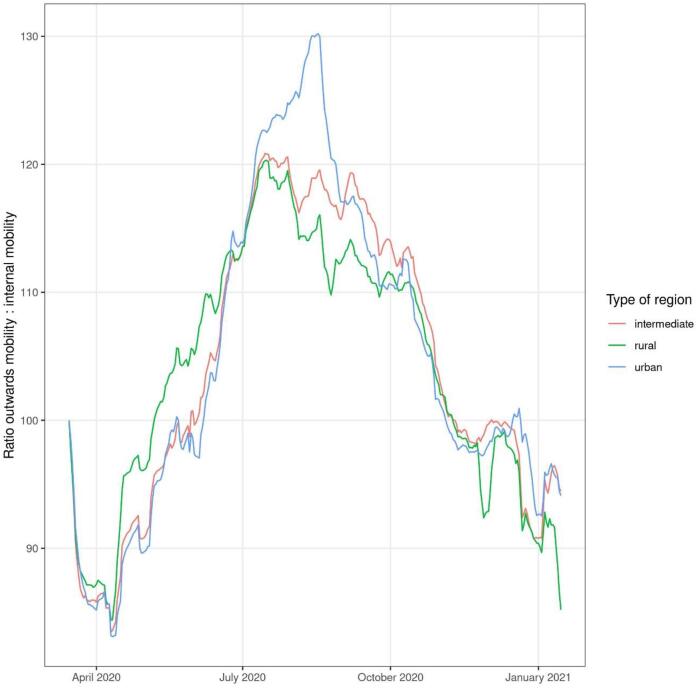
Ratio of outwards mobility indicator to internal mobility indicator, 7-day moving average, in % with respect to internal mobility indicator.

Nevertheless, the trend of inwards/ outwards mobility differs from the one of internal mobility. The number of trips outside each NUTS3 zone decreased significantly more than trips within each NUTS3 zone during the first wave of the pandemic. As Fig. 7 shows, the mean ratio between the outwards and the internal mobility indicators rapidly fell to below 85% on average by mid-April 2020 for all regional typologies. Once the measures restricted mobility across regions were lifted in the majority of EU Member States (May 2020), the ratio recovered fast towards 100% (i.e. internal and outwards mobility levels returned to their pre-pandemic relative weights). During the summer the ratio was reversed and the relative weight of outwards (and -due to the symmetry- also of inwards) mobility increased to 130%. The high value for urban areas confirms the hypothesis of residents in urban areas moving to non-urban areas during the summer. The ratio gradually fell back to the 100% level in autumn, with the urban areas consistently having a lower ratio than non-urban areas.

The measures applied in November and December 2020 to limit the evolution of the second wave of the pandemic included the restriction of mobility between regions in several EU Member States. They had a clear impact on the ratio of outwards mobility, which reached levels comparable of those during the first wave in March-April 2020. Throughout the period March-December 2020, it is evident that fluctuations in the level of mobility were the result of the restrictiveness of policy measures to fight the pandemic.

### 4.2 Correlation of mobility with spread of pandemic at regional level

While the effectiveness of non-pharmaceutical measures in limiting mobility is easy to demonstrate, the impact on controlling the evolution of the pandemic is less straightforward. There are several indicators for the evaluation of the epidemiological situation at country and –in some cases- region level, including the measurement of daily and/or accumulated reported cases, hospitalizations, intensive care patients or deaths related to Covid-19. The latter may be the most reliable measure of the true impact of the pandemic in terms of human lives, but is also the result of a combination of numerous confounding variables that limit the ability to identify clear cause and effect relationships. For example, the number of Covid-19 related deaths in a specific region given a certain share of contagion in the population probably depends more on the local health system conditions, response mechanisms and demographics rather than on the virus propagation dynamics themselves.

Since we are addressing the contribution of mobility on the dynamics of the pandemic, the most suitable indicator for the exploration of causality would be the number of infected individuals, a possible direct effect of social interaction and –by extension- mobility. The disadvantage of this measure is –however- its lower reliability. Especially during the early phases of the pandemic, the number of cases was seriously under-reported since the number of tests was limited and the sampling was biased. The testing strategies and –in consequence- the reporting quality improved by June 2020, even though still at varying degrees across countries. As a specific indicator, we use the 14-day notification rate of new Covid-19 cases per 100 000 inhabitants, one of the most frequently used indicators for reporting the pandemic’s evolution (European Centre for Disease Prevention and Control, 2020). The data available from the Covid-19 tracker (Naqvi, 2021a, Naqvi, 2021) have been collected through a combination of regional, national and international sources, ensuring that they are consistent at country level and in line with the data reported at European level. The notification rate at regional level is the highest spatial resolution data source that allows comparisons across the EU.

**Fig. 8:**
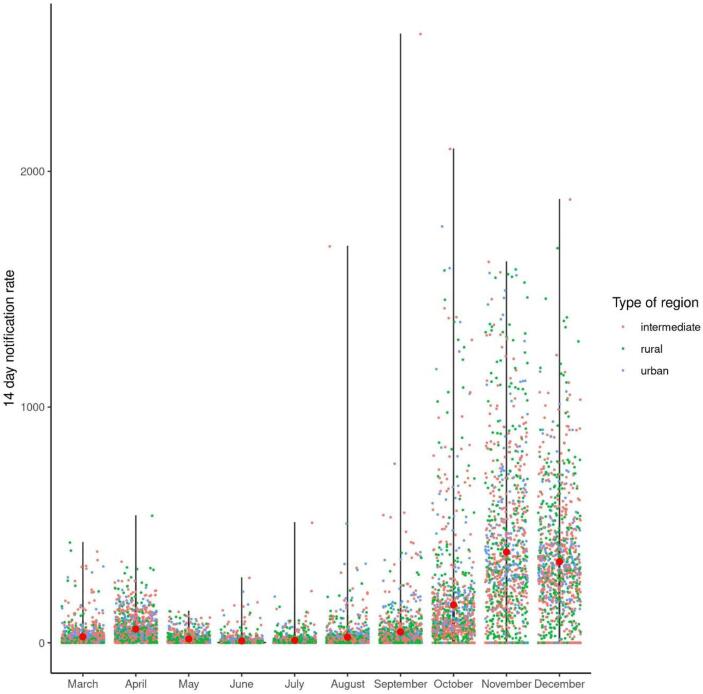
14-day notification rate of Covid-19 reported cases per 100 000 inhabitants at NUTS 3 region level.

Visualizing the notification rate at regional level over time (Fig. 8) suggests that its variance was relatively low after the first wave (May 2020), gradually increased during the summer months, and reached maximum level in the height of the second wave (November- December 2020). This pattern reflects a high concentration of Covid-19 incidence in a few regions in April-May 2020 and –in contrast- a wide spatial dispersion after September 2020. It is also interesting to note that there is no apparent pattern in terms of a specific urbanization profile consistently showing a high incidence.

If mobility and urbanization typology are both taken into account, however, the interpretation of the correlations changes. We used a simple observational model to explore a possible link between increased levels of mobility and high levels of Covid-19 incidence. We grouped the regions in the dataset according to their mean mobility indicator during July and August 2020, for all three types of mobility (internal, inwards, outwards), and defined regions as having had high levels of mobility for any of the three types if the respective mobility indicator was over 90%. In a similar fashion, we grouped the regions in terms of their level of Covid-19 incidence in October 2020. We considered as regions of high incidence the ones that had an average 14-day notification rate in the 80% percentile within their country.

This formulation allows us to test whether increased levels of mobility in a region eventually led to high numbers of reported Covid-19 cases, with the region population already accounted for. In addition, we combined the level of mobility and the urban typology of each region and created 6 different region categories (3 urbanization profiles × 2 mobility levels).

**Fig. 9:**
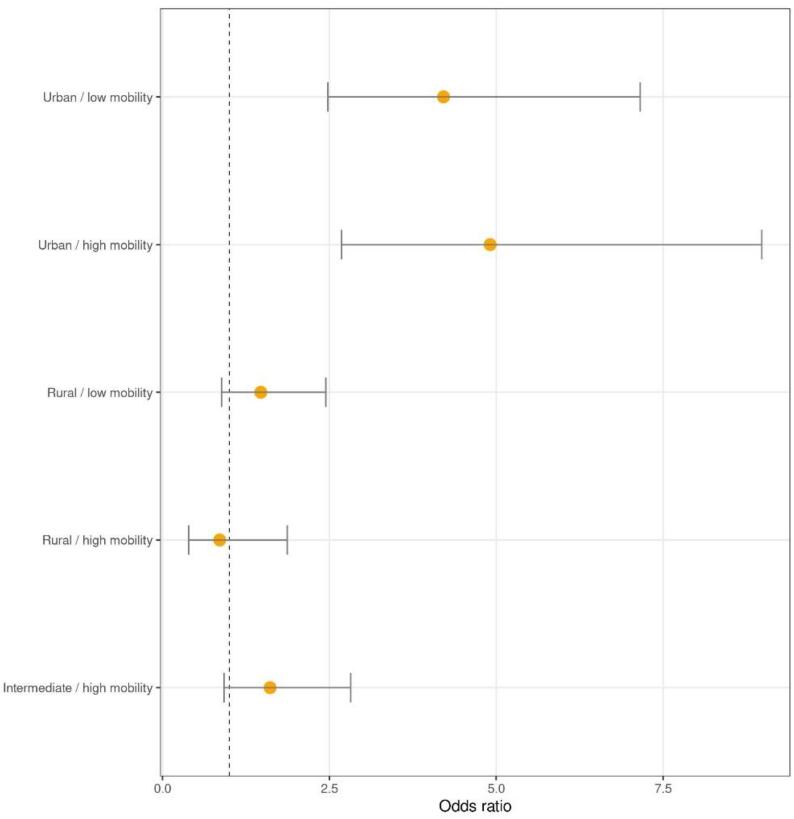
Odds ratios of high Covid-19 notification rate in October 2020, internal mobility (in relation to intermediate urbanization zones with low internal mobility levels in July-August 2020).

**Fig. 10:**
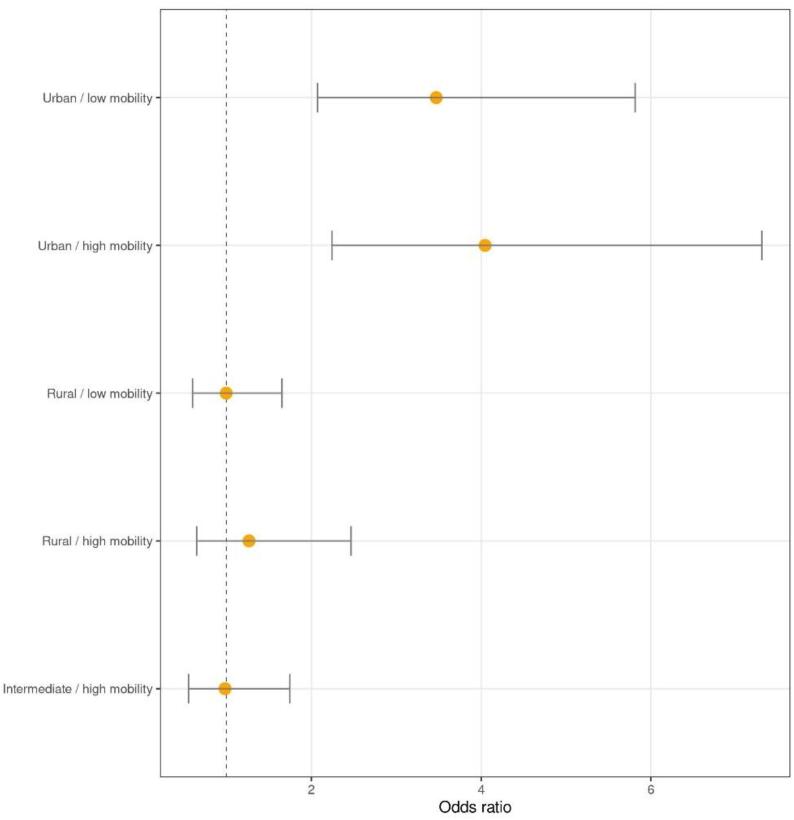
Odds ratios of high Covid-19 notification rate in October 2020, inwards mobility (in relation to intermediate urbanization zones with low inwards mobility levels in July-August 2020).

**Fig. 11:**
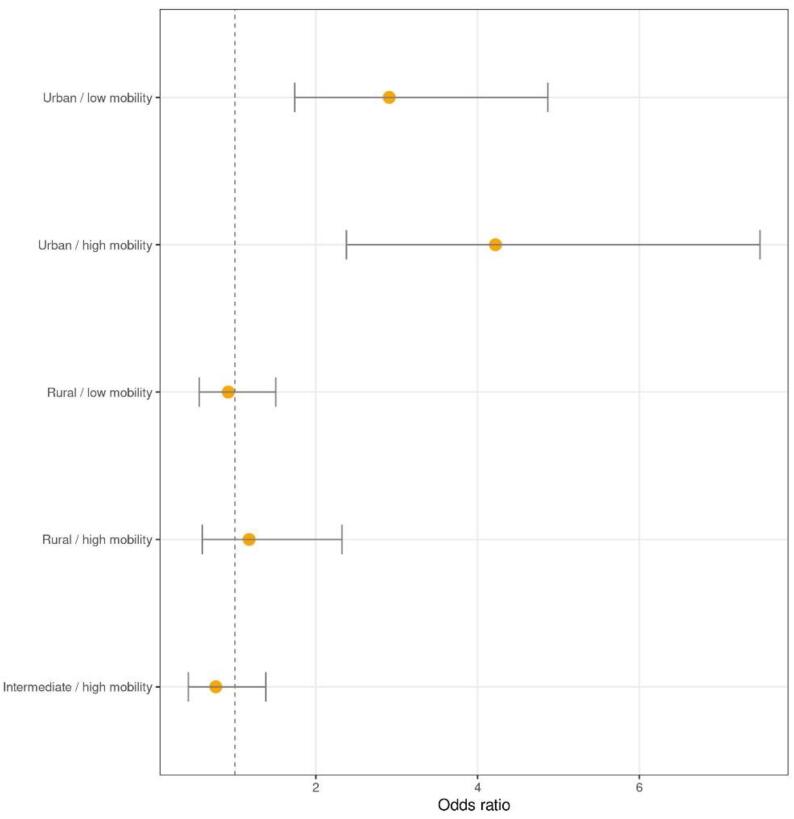
Odds ratios of high Covid-19 notification rate in October 2020, outwards mobility (in relation to intermediate urbanization zones with low outwards mobility levels in July-August 2020).

The main test we performed was the comparison of the odds ratios of the six categories as regards the probability for a region to present a high incidence level in October 2020. We compared the odds of each category to those of the regions with an intermediate profile and low mobility. Fig. 9, Fig. 10, Fig. 11 visualize the odds ratios at a 95% confidence level for internal, inwards and outwards mobility respectively.

The mean odds ratios and the width of their confidence intervals provide a quantitative estimate of the relative probabilities. For internal mobility (Fig. 9), the odds of an urban area to report a higher number of cases per unit of population are unambiguously higher than those of non-urban regions, regardless of the level of mobility. Urban areas appear to have at least 2.5 times higher odds than an intermediate region with low mobility during July and August 2020 to have a high Covid-19 incidence in October 2020. For urban regions that had high mobility levels in the summer of 2020, the odds are even higher. On average, they show a 5:1 odds ratio compared to the base case (intermediate region with low mobility), significantly higher than the already high 4:1 odds ratio of urban regions with low internal mobility levels. In both cases though, the range of the confidence interval is very wide, a result of the high variability of the evolution of the pandemic among urban regions in the EU. The impact of high internal mobility levels is also evident in the case of intermediate urbanization regions. The odds are –on average- higher than in intermediate zones with low mobility at a high level of confidence. In contrast, the odds ratios for rural areas give an unclear message. Whereas one would expect rural areas with low mobility levels to have lower odds than intermediate regions with low mobility levels, this is not the case. Moreover, increased internal mobility levels appear as leading to a decrease in odds ratios. These counter-intuitive cases may be explained by the non-uniform distribution of rural regions across countries with high Covid-19 prevalence.

A similar picture overall can be drawn for inwards and outwards mobility (Fig. 10, Fig. 11). The majority of the odds ratios are in line with expectations and urban areas have in both cases higher probabilities to have registered a high rate of cases in October 2020. Increased mobility levels during the summer further increase these odds. The average odds ratio for the other categories is close to 1:1 and the impact of high mobility levels is visible but highly uncertain.

Notwithstanding the few unexpected results, we may still draw a conclusion that can be generalized at EU level. Urban areas tend to have a higher rate of Covid-19 cases and increased levels of mobility accentuate the urbanization effect. Both aspects reinforce the importance of population density and volume of social interaction as a factor of the disease propagation. In that sense, mobility should be seen as a means to facilitate social interaction rather than a driver itself. Movements between regions do not appear to have significantly accelerated the evolution of the pandemic in non-urban regions. We can therefore not claim any direct regional spillover effect due to increased mobility. On the other hand, we cannot discard another possible mechanism that could still explain most of the observations. The increase in mobility levels may be primarily an expression of the general increase in social interaction that -combined with the overall relaxation of personal protection measures- led to a new wave when the majority of the residents of urban areas returned after the summer holidays.

## 5 Conclusions

Several Mobile Network Operators have been providing aggregate and anonymized mobility data at fine spatial and temporal resolution in order to assist policy makers in monitoring the evolution of mobility during the Covid-19 pandemic. The analysis of Mobile Network Operator data at regional level (NUTS3) reveals varying patterns depending on the temporal evolution of the pandemic in each EU Member State, the measures taken at local or national level to limit the growth of the pandemic, as well as the level of urbanization and type of economic activity in each region.

During the first phase of the pandemic (March- April 2020) the decrease in mobility was in general uniform among regions in the same Member State, especially in Italy, Spain and France, where national level measures were adopted. A relaxation of the measures and a resulting rebound of mobility was evident during the summer period (July- August 2020). At the same time, a shift from urban to rural areas during the summer vacation period is evident, with especially touristic areas increasing the number of movements in the same Member State. The variance in mobility trends during the second wave of the pandemic (October- November 2020) was higher, a result of the predominantly local and regional level measures applied in each Member State. Those insights suggest a certain correlation between the level of mobility and the evolution of the pandemic at regional level. The combination of mobile phones and epidemiological data at regional level reveals that urban regions are more prone to reaching high levels of Covid-19 prevalence. If accompanied by high levels of mobility, the epidemiological risk for urban regions becomes even higher. Nevertheless, since intra-zonal and inter-zonal mobility are only two of the numerous factors that affect the geographic distribution of the pandemic (Christidis and Christodoulou, 2020), it would be premature to establish a causation effect.

The results suggest that there is a high degree of variability in the evolution of mobility and prevalence at regional level during the pandemic. The decrease in mobility was more evident in urban areas when country-wide restrictions were in place, presumably due to the higher share of activities that can be replaced with tele-working. Urban areas also show a decline in mobility during weekends and holidays, owing to the significant share of employment and education in their normal levels of activity. Non-pharmaceutical interventions are the primary driver for decreased mobility throughout the EU. Voluntary confinement appears to have played a role during the first wave of the pandemic, but gradually relaxed –as did general compliance to restrictions- until the second wave was evident. Mobility levels in non-urban areas tended to recover faster than in urban areas, possibly due to the increased movement of travellers from urban areas.

Given the persevering nature of the pandemic and the considerable possibility of further waves in the future, a main lesson drawn from the analysis of dynamics of intra- and inter-regional mobility concerns the question of restricting movements between regions. The new waves in the pandemic in Europe following the holiday periods in summer and New Year appear to be a repercussion of the activity in the destination regions and not of increased mobility per se. Measures aiming to limit future contagions should therefore prioritize limiting unsafe interactions among the population before restricting travel options. Future work, once a better understanding of the Covid-19 transmission mechanism is achieved, can produce improved models of the role of mobility and regional profile and contribute in the identification of more effective prevention policies.

### CRediT authorship contribution statement

**Panayotis Christidis:** Conceptualization, Data curation, Formal analysis, Methodology, Writing – original draft, Writing – review & editing. **Biagio Ciuffo:** Conceptualization, Formal analysis, Methodology, Writing – review & editing. **Michele Vespe:** Conceptualization, Formal analysis, Methodology, Writing – review & editing.

## References

[b0005] Bwambale A., Choudhury C.F., Hess S. (2019). Modelling trip generation using mobile phone data: A latent demographics approach. J. Transp. Geogr..

[b0010] Chang S., Pierson E., Koh P.W., Gerardin J., Redbird B., Grusky D., Leskovec J. (2021). Mobility network models of COVID-19 explain inequities and inform reopening. Nature.

[b0015] Christidis P., Christodoulou A. (2020). The predictive capacity of air travel patterns during the global spread of the covid-19 pandemic: Risk, uncertainty and randomness. Int. J. Environ. Res. Public Health.

[b0020] Christidis P., Christodoulou A., Navajas-Cawood E., Ciuffo B. (2021). The Post-Pandemic Recovery of Transport Activity: Emerging Mobility Patterns and Repercussions on Future Evolution. Sustainability.

[b0025] European Centre for Disease Prevention and Control, 2020. Updated projections of COVID-19 in the EU/EEA and the UK. 23 November 2020.

[b0030] European Commission, 2020. Recommendation (EU) 2020/518 of 8 April 2020 on a common Union toolbox for the use of technology and data to combat and exit from the COVID-19 crisis, in particular concerning mobile application and the use of anonymised mobility data, Recommendation (EU).

[b0035] EUROSTAT, n.d. Population change - Demographic balance and crude rates at regional level (NUTS 3) (demo_r_gind3).

[b0040] Fiorello D., Martino A., Zani L., Christidis P., Navajas-Cawood E. (2016). Mobility Data across the EU 28 Member States. Results from an Extensive CAWI Survey.

[b0045] Focas, C., Christidis, P., 2017. Peak Car in Europe? Presented at the Transportation Research Procedia, pp. 531–550. https://doi.org/10.1016/j.trpro.2017.05.437.

[b0050] Gao S., Rao J., Kang Y., Liang Y., Kruse J., Dopfer D., Sethi A.K., Mandujano Reyes J.F., Yandell B.S., Patz J.A. (2020). Association of Mobile Phone Location Data Indications of Travel and Stay-at-Home Mandates With COVID-19 Infection Rates in the US. JAMA network open.

[b0055] GISCO, NUTS 2016 definition, n.d.

[b0060] Kang Y., Gao S., Liang Y., Li M., Rao J., Kruse J. (2020). Multiscale dynamic human mobility flow dataset in the U.S. during the COVID-19 epidemic. Scientific Data.

[b0065] Kishore N., Kiang M.V., Engø-Monsen K., Vembar N., Schroeder A., Balsari S., Buckee C.O. (2020). Measuring mobility to monitor travel and physical distancing interventions: a common framework for mobile phone data analysis. Lancet Digital Health.

[b0070] Naqvi, A., 2021a. COVID-19 European regional tracker. medRxiv. https://doi.org/10.1101/2021.02.15.21251788.

[b0075] Naqvi A. (2021). asjadnaqvi/COVID19-European-Regional-Tracker: COVID-19 European Regional Tracker - June 2021. Zenodo.

[b0080] Pullano G., Valdano E., Scarpa N., Rubrichi S., Colizza V. (2020). Evaluating the effect of demographic factors, socioeconomic factors, and risk aversion on mobility during the COVID-19 epidemic in France under lockdown: a population-based study. Lancet Digital Health.

[b0085] Santamaria,C., Sermi, F., Spyratos, S., Iacus, S., Annunziato, A., Tarchi, D., Vespe, M., 2020. Measuring the Impact of COVID-19 Confinement Measures on Human Mobility using Mobile Positioning Data (Technical Report No. JRC121298). Publications Office of the European Union, JRC121298, Luxembourg. https://doi.org/10.2760/913067.

[b0090] Schlosser F., Maier B.F., Jack O., Hinrichs D., Zachariae A., Brockmann D. (2020). COVID-19 lockdown induces disease-mitigating structural changes in mobility networks. Proc. Nat. Acad. Sci. U.S.A.

[b0095] López Soler J.R., Christidis P., Vassallo J.M. (2021). Teleworking and online shopping: Socio-economic factors affecting their impact on transport demand. Sustainability (Switzerland).

[b0100] Vespe, M., Minora, U., Iacus, S.M., Spyratos, S., Sermi, F., Fontana, M., Ciuffo, B., Christidis, P., 2021. Mobility and Economic Impact of COVID-19 Restrictions in Italy using Mobile Network Operator Data.

[b0105] Yabe T., Tsubouchi K., Fujiwara N., Wada T., Sekimoto Y., Ukkusuri S.V. (2020). Non-compulsory measures sufficiently reduced human mobility in Tokyo during the COVID-19 epidemic. Sci. Rep..

